# Estimation of the Effect of Salt-Intake Reduction on Cardiovascular Mortality Decline between 1950 and 2017 in Japan: A Retrospective Simulation Study

**DOI:** 10.3390/nu14183747

**Published:** 2022-09-10

**Authors:** Takehiro Sugiyama, Nayu Ikeda, Kazuko Minowa, Nobuo Nishi

**Affiliations:** 1 Diabetes and Metabolism Information Center, Research Institute, National Center for Global Health and Medicine, Tokyo 162-8655, Japan; 2 Institute for Global Health Policy Research, Bureau of International Health Cooperation, National Center for Global Health and Medicine, Tokyo 162-8655, Japan; 3 Department of Health Services Research, Faculty of Medicine, University of Tsukuba, Tsukuba 305-8575, Ibaraki, Japan; 4 International Center for Nutrition and Information, National Institute of Health and Nutrition, National Institutes of Biomedical Innovation, Health and Nutrition, Tokyo 162-8636, Japan

**Keywords:** system analysis, sodium chloride, cardiovascular diseases, mortality, National Health and Nutrition Survey, Japan

## Abstract

In Japan, a decrease in cardiovascular mortality has coincided with reduced population salt intake since the 1950s. The purpose of this study was to quantify the effect of reduced population salt intake on the long-term trends of cardiovascular mortality. Using government statistics and epidemiological study results in people of 20–69 years old from 1950 to 2017, including the National Health and Nutrition Survey, we developed a system dynamics model of age-specific cardiovascular mortality and salt intake. We estimated the period and cohort effects on mortality and calibrated the model for the historical mortality rate. We then simulated the counterfactual scenario of no decrease in salt intake to estimate the reduction in cardiovascular deaths associated with decreased mean salt intake. Compared with the base run and calibrated to the actual data, approximately 298,000 and 118,000 excess deaths were observed in men and women, respectively, assuming no change in salt intake over the entire period. The model suggests that the decline in salt intake since the 1950s has contributed to a non-negligible reduction in cardiovascular mortality.

## 1. Introduction

Cardiovascular disease is the leading cause of death and disability worldwide [1]. In Japan, age-adjusted death rates from stroke have declined dramatically since the mid-1960s, and those from ischemic heart disease have slowly decreased over time [2]. These improvements in cardiovascular mortality occurred almost concurrently with reductions in population-level blood pressure and dietary salt intake [3,4]. The decline in cardiovascular mortality over time may be partly attributed to blood-pressure lowering caused by the reduction in dietary salt intake in the population [5,6,7].

The link between salt intake and cardiovascular disease has been shown in previous studies. Hypertension is a well-known risk factor for cardiovascular diseases; the international study of salt and blood pressure (INTERSALT) showed a positive relationship between the amount of salt intake and blood pressure [8]. A meta-analysis of prospective studies concluded that higher salt intake is associated with an increased risk of total cardiovascular disease, especially stroke [9]. A meta-analysis of intervention studies [10]—a re-analysis of a similar previous study that showed non-significant results [11]—illustrated the effect of salt reduction on the prevention of cardiovascular events. According to a modeling study, sodium intake exceeding the guideline amount of 2.0 g per day contributed to 1,650,000 deaths from cardiovascular disease in 2010 [12]. Furthermore, food selection is an important determinant of the amount of salt intake. In Japan, primary food sources of sodium are soy sauce, salted vegetables, soups (e.g., miso soup), salted fish, seafood, and fish roe [13]. The dietary approaches to stop hypertension (DASH) diet contributed to decreased blood pressure through sodium intake reduction and potassium intake increase [14]. At the 24-year follow-up, middle-aged women who had followed a DASH-style diet had a decreased risk of coronary heart disease and stroke [15]. In keeping with the evidence above, guidelines in Japan recommend further salt-intake reduction (6.0 g per day or lower for hypertensive patients, 7.5 g per day for adult men, and 6.5 g per day for adult women) [16,17]. Guidelines in other countries and the World Health Organization [18] recommend stricter restrictions.

However, the level of reduction in population-level salt intake that has contributed to the decline in cardiovascular mortality over the past several decades has not yet been quantified. A previous study attributed nearly 25% of the decline in coronary heart disease mortality rates between 1980 and 2012 to the decrease in blood pressure [19]. Another study decomposed the changes in cerebrovascular mortality from 1920 to 2003 by age, period, and cohort effect [20]. Although these previous studies referred to reduced salt intake as a potential distal factor, the quantitative impact of decreased salt consumption on the decline in cardiovascular mortality rates remains unclear.

We aimed to examine the magnitude of the effect of population-level dietary salt reduction on the prevention of cardiovascular mortality from the postwar period to the present in Japan. We developed a system dynamics model to conduct a retrospective simulation of the changes in cardiovascular mortality and salt intake and incorporated the concept of the decomposition of age–period–cohort effects.

## 2. Materials and Methods

### 2.1. Study Overview

We estimated the effect of reduced population-level dietary salt intake on the decline of cardiovascular mortality in Japanese adults aged 20 to 69 years in the period after World War II. We set a time frame of 1950 to 2017 for the simulation. We did not include cardiovascular death at 70 years of age and older because (1) data on some input parameters were unavailable by age in 10-year or smaller bins and (2) we aimed to focus on working-age groups in which preventing mortality is especially important. We excluded those younger than 20 years because cardiovascular death is rare in this age group.

### 2.2. Model Structure

**Figure 1:**
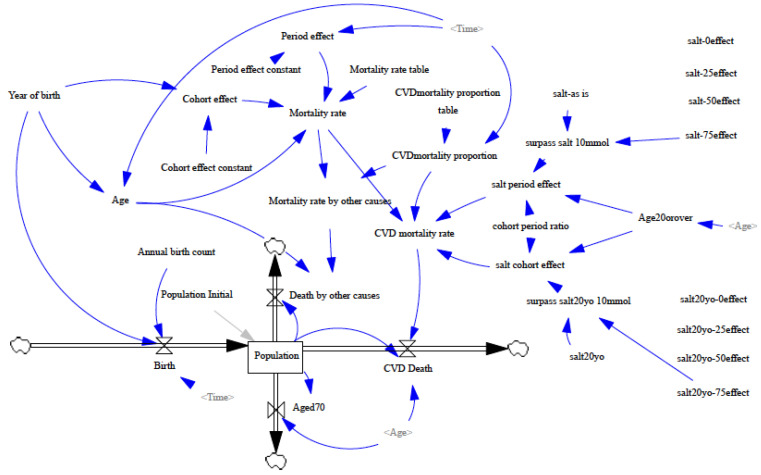
System dynamics model of the age–period–cohort effects of dietary salt intake on cardiovascular mortality in the Japanese adult population aged 20 to 69 years between 1950 and 2017.

We constructed a system dynamics model [21] using Vensim^®^ DSS for Macintosh v8.2.1 Double Precision x64 (Ventana Systems, Inc. Harvard, MA, USA). Figure 1 provides an overview of the system dynamics model. The model comprises the stock-and-flow structure of population dynamics and the effect mechanism of dietary salt intake on cardiovascular death by age, period, and cohort. We developed the model separately by sex and replicated the model for each of the 118 cohorts born between 1880 and 1997, thereby covering the population aged 20 to 70 years between 1950 and 2017.

In the stock-flow diagram part of the model, the stock variable in the box represents the population aged between 0 and 70 years (Population; Figure 1). Individuals enter the stock variable as inflows at birth (Birth). Individuals exit the stock variable as outflows when they die of cardiovascular disease (CVD Death) or other causes (Death by other causes) or when they reach their 70th birthday (Aged 70).

Although we adopted the concept of age–period–cohort effect decomposition, we imposed stricter conditions on the model than we did in previous studies [20,22]. We modeled the overall mortality rate as the product of age, period, and cohort effects. Specifically, we first identified the mortality rate for each age group in 1950 (Mortality rate table, which represents age effect). Then, we multiplied the mortality rate by parameter p, which models the exponential decrease in mortality since 1950 (period effect), and by parameter c, which increases (or decreases) by a factor of c for each additional year of cohort membership (cohort effect). In other words, we modeled the exponential decay in mortality rates since 1950 as the period effect and the decreases for each additional year of cohort membership as the cohort effect.

We decomposed the mortality rate into cardiovascular mortality rate and mortality rate by other causes, multiplying the proportion of cardiovascular mortality for each period. We modeled mortality from other causes to ensure it was unaffected by differences in salt intake. In contrast, we modeled cardiovascular mortality to ensure it was influenced by the cohort and age effects of salt intake. These effects were considered when simulating scenarios of salt-intake reduction.

### 2.3. Input Parameters and Data

We used published data to prepare a dataset of population, births, mortality rates, and average dietary salt intake by single year of age and sex in the study population from birth until 2017 or the age of 70 years. Table 1 lists the sources of published data used in this study.

We established initial population values by single year of age and sex as of 1950 for cohorts born in 1949 or earlier. For cohorts born from 1950 onward, we added the number of births as the inflow into the population in each cohort’s birth year. We used the mortality rate for each age in 1950 as the age effect when modeling the age- and period-specific mortality rate for each cohort. We introduced data on cardiovascular mortality as a proportion of overall mortality from Vital Statistics [24]. While cardiovascular mortality as a proportion of overall mortality should differ by age and period, we assumed a universal cardiovascular mortality rate across periods given our information was restricted to the cause of death for all ages.

**Figure 2:**
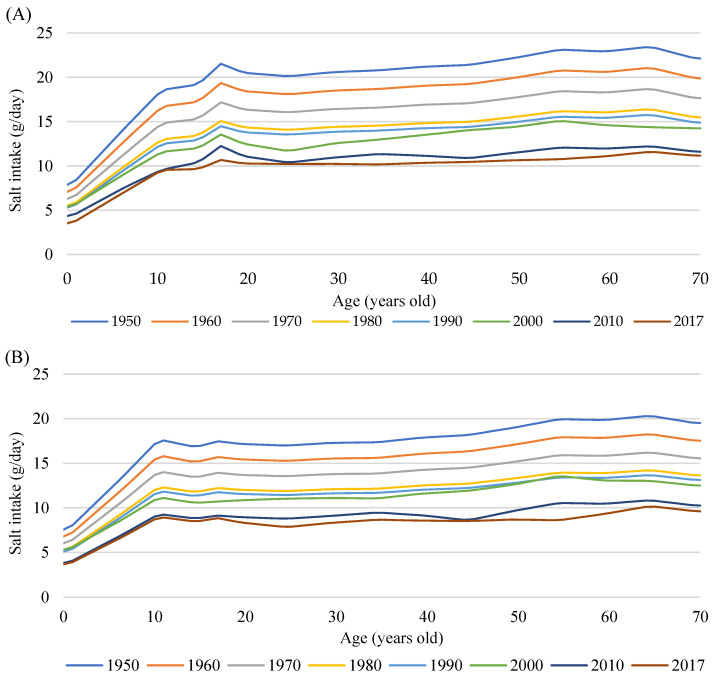
Age-specific average salt intake in selected years in men (**A**) and women (**B**), estimated from National Nutrition Surveys between 1973 and 2002 and National Health and Nutrition Surveys between 2003 and 2017.

We obtained published data on average salt intake from the National Nutrition Surveys between 1973 and 2002 and the National Health and Nutrition Surveys (henceforth both referred to as the NHNS) between 2003 and 2017 [26]. The NHNS’s methodological details have been described elsewhere [27,28]. Briefly, the NHNS is an annual cross-sectional household interview and examination survey conducted with a nationally representative sample of individuals aged 1 year and older. Dietary intake data were collected at the household level until the survey began recording individual-level intake data in 1995. Therefore, annual data on average salt intake are available as only overall averages between 1973 and 1994 and as averages by 10-year age group and sex between 1995 and 2017. We assigned the same values to all ages within each sex and 10-year age group from 1995 to 2017. To impute missing values for average dietary salt intake by age and sex for the years 1994 and earlier, we first calculated the overall annual averages between 1950 and 1972 by linear extrapolation of the values from 1973 to 1986, demonstrating a relatively linear trend (Supplementary Figure S1). We placed a cap on extrapolated values exceeding 20 g for males in 1942 and earlier by referencing a previous study conducted in the prewar period [29]. To further compute average salt intake by age and sex for the years 1994 and earlier, we multiplied the overall average in each year by the ratio of the arithmetic mean of averages in each sex and age group to the arithmetic mean of the overall averages for the period of 1995 to 2017. Figure 2 illustrates the estimated average dietary salt intake by age and sex over time.

To assess the effect of salt intake, we applied a previous study’s finding indicating that an increase in daily salt intake by 10 mmol (0.584 g) was associated with a 1% increase in cardiovascular mortality [30]. We divided the effect of salt intake on mortality into the period and cohort effects by a ratio of 1 to 1. This ratio was adopted based on the assumption that the average salt intake to date affects cardiovascular mortality through elevated blood pressure and atherosclerosis.

We derived the period effect as the difference between the simulation’s assumed and actual salt intake at a given time. In contrast, the cohort effect was derived as the difference between the assumed and actual salt intake at the age of 20 years for that cohort. We applied these effects only to individuals aged 20 years or older.

### 2.4. Model Optimization (Calibration) and Simulation Scenarios

To optimize our system dynamics model, we calibrated mortality rates estimated from the model with actual mortality rates and established the base-run factual scenario. Through model calibration, we obtained the coefficients of the period and cohort effects of factors other than salt intake—such as progress in treatment for hypertension and improvement in emergency medicine. Holding these coefficients constant, we simulated four counterfactual scenarios, whereby the effects of population-level dietary salt reduction on cardiovascular mortality were 75%, 50%, 25%, and 0% of the actual effects since 1950. We compared the estimated excess cardiovascular mortality between the base run and counterfactual scenarios to examine the effect of salt-intake reduction.

## 3. Results

### 3.1. Simulation of Cardiovascular Mortality Rate

**Figure 3:**
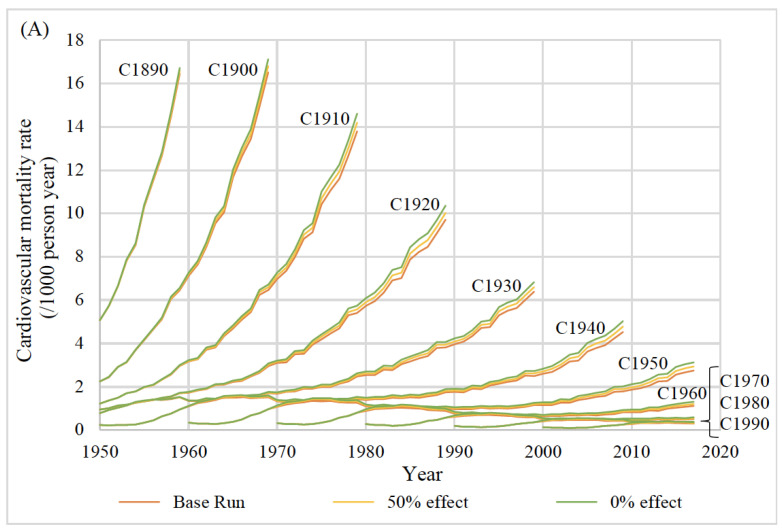

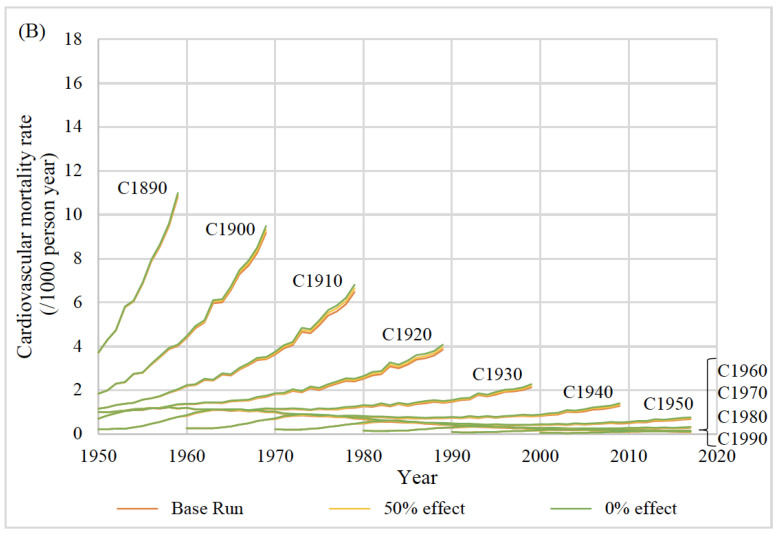
Estimated cardiovascular mortality rates in selected birth cohorts in men (**A**) and women (**B**). The numbers following the letter “C” indicate birth years (e.g., C1890 represents the 1890 birth cohort). Base run: the actual effect of salt-intake reduction on the cardiovascular mortality rate; 50% effect: 50% of the actual effect of salt-intake reduction; 0% effect: no effect of salt-intake reduction since 1950.

The coefficients of period and cohort effects were estimated at 0.97777 and 0.999826, respectively, in men and at 0.962632 and 0.998483, respectively, in women. Figure 3 shows the changes in the estimated cardiovascular mortality rates over time for selected birth cohorts and scenarios. The cardiovascular mortality rates were lower in more recent birth cohorts when compared at the same age (e.g., the right end of each curve represents the mortality rate at the age of 69 years). Moreover, the difference between the base run and counterfactual scenarios widened over time. The absolute difference was larger in less recent cohorts whereas the relative difference was more significant in more recent cohorts. For example, for the 1910 birth cohort in the year 1979 (e.g., at the age of 69 years), the absolute and relative differences in the cardiovascular mortality rate between the base run and the scenario of null effects were 0.81/1000 person-years and 5.9%, respectively, in men and 0.33/1000 person-years and 5.1%, respectively, in women. For the 1940 birth cohort in the year 2009, these differences were 0.50/1000 person-years and 11.1%, respectively, in men and 0.13/1000 person-years and 9.8%, respectively, in women.

### 3.2. Excess Cardiovascular Mortality by Year

**Figure 4:**
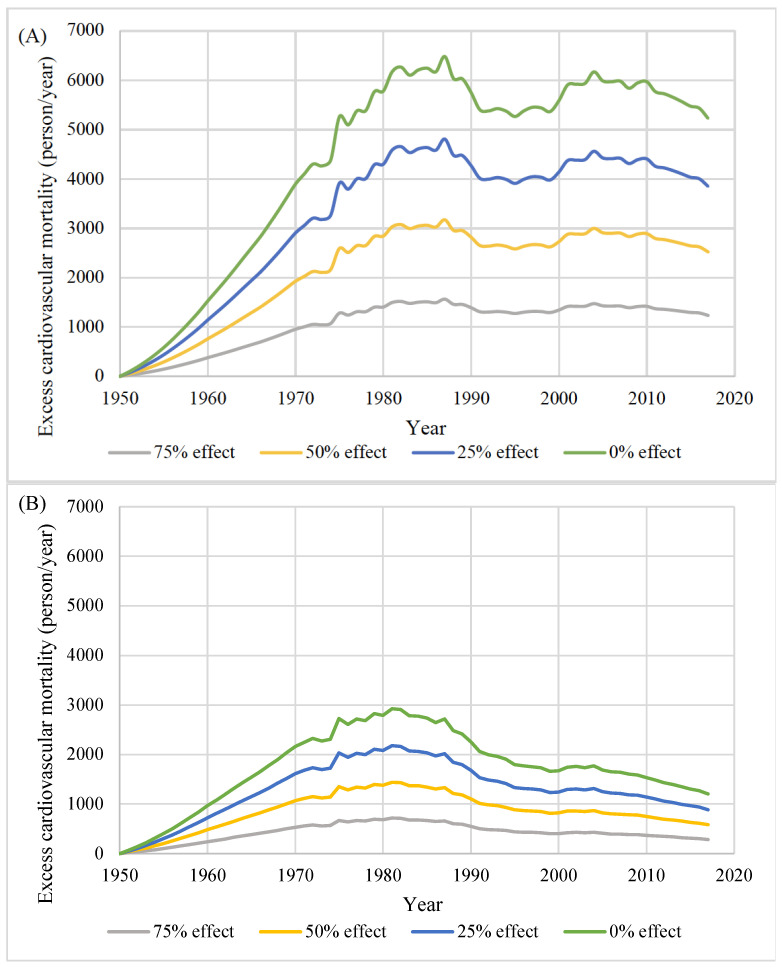
Absolute excess cardiovascular mortality by scenario in men (**A**) and women (**B**): 75% effect, 75% of the actual effect of salt-intake reduction; 50% effect, 50% of the actual effect of salt-intake reduction; 25% effect, 25% of the actual effect of salt-intake reduction; 0% effect, no effect of salt-intake reduction since 1950.

**Figure 5:**
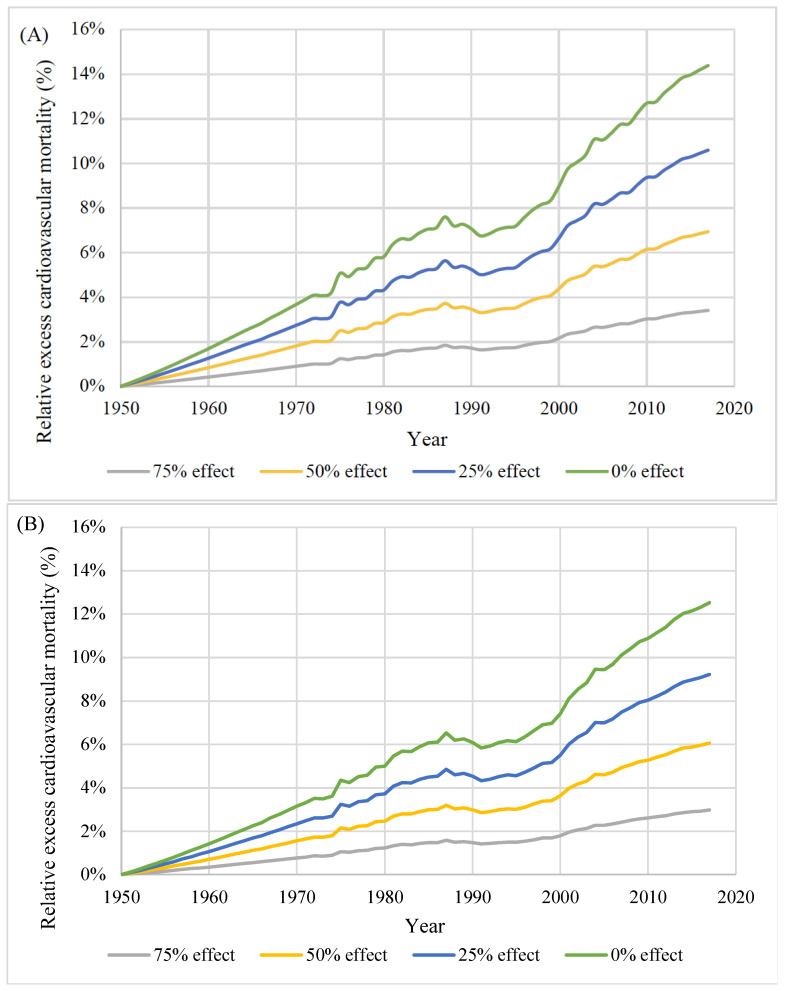
Relative excess cardiovascular mortality by scenario in men (**A**) and women (**B**): 75% effect, 75% of the actual effect of salt-intake reduction; 50% effect, 50% of the actual effect of salt-intake reduction; 25% effect, 25% of the actual effect of salt-intake reduction; 0% effect, no effect of salt-intake reduction since 1950.

Figure 4 and Figure 5 show the changes in the absolute and relative excess cardiovascular mortality over time in the counterfactual scenarios compared with the base run. For men, the absolute excess mortality increased until the 1980s; then, the trend plateaued. Excess mortality was the highest in 1987 (e.g., 6476 persons/year in the 0% effect scenario). For women, the absolute excess mortality was the highest in 1981 (e.g., 2922 in the 0% effect scenario) and consistently declined. The relative excess cardiovascular mortality almost consistently increased over time, with a temporary plateau around 1990; the largest relative increase was observed toward the end of the observation period, reaching 14.4% in men and 12.5% in women in 2017 in the 0% effect scenario.

### 3.3. Accumulated Excess Cardiovascular Mortality during the Observation Period

**Figure 6:**
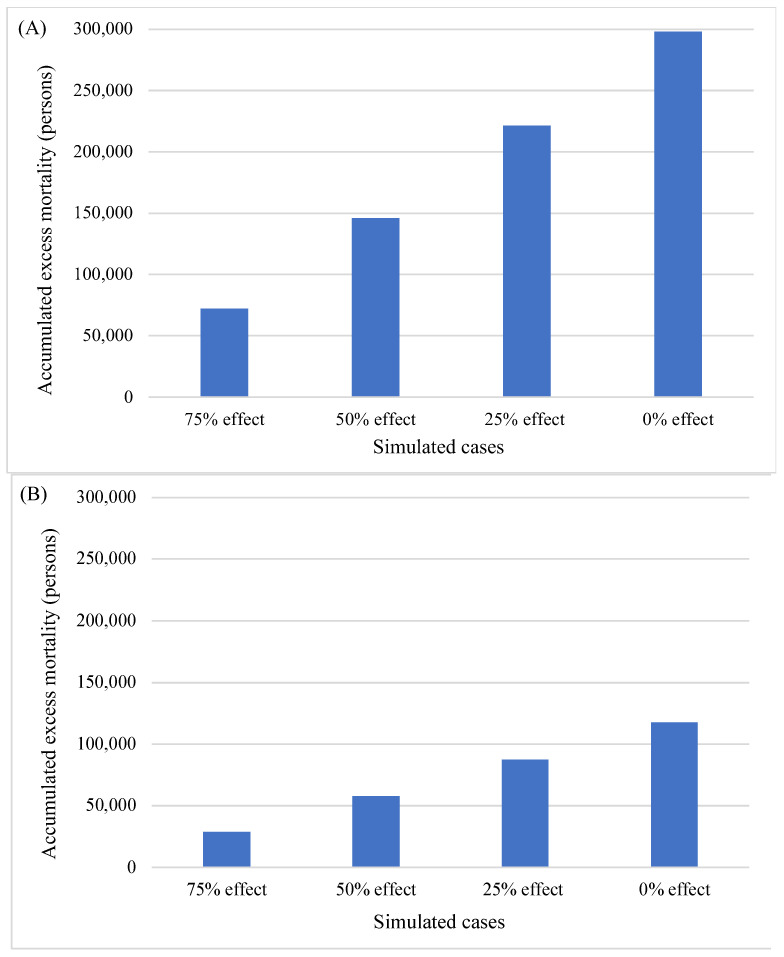
Accumulated excess cardiovascular mortality (number of persons) between 1950 and 2017. (**A**) Men and (**B**) women: 75% effect, 75% of the actual effect of salt-intake reduction; 50% effect, 50% of the actual effect of salt-intake reduction; 25% effect, 25% of the actual effect of salt-intake reduction; 0% effect, no effect of salt-intake reduction since 1950.

Figure 6 illustrates the accumulated excess mortality in the simulated models. Assuming no change in salt intake, we estimated 298,000 excess deaths of men and 118,000 excess deaths of women throughout the study period. The salt-intake reduction effect was nearly linear; in the 75% salt-intake reduction scenario, we estimated 72,000 and 29,000 excess deaths in men and women, respectively.

## 4. Discussion

This study used a system dynamics model to examine the impact of reduced salt intake on cardiovascular mortality, focusing on the effect in the relatively low mortality group of people aged 20 to 69 years old. We illustrated that the trend of absolute excess cardiovascular mortality plateaued in men and declined in women. In contrast, the trend of relative excess cardiovascular mortality consistently increased in men and women. We estimated approximately 298,000 and 118,000 excess premature deaths of men and women throughout the observation period, assuming no change in salt intake over the period. To our knowledge, this is the first study to estimate the effect of salt-intake reduction on cardiovascular mortality in Japan’s postwar period.

Miwa et al. estimated the magnitude of age, period, and cohort effects on cerebrovascular disease mortality to clarify the factors contributing to the changes in cerebrovascular disease mortality during the 20th century [20]. Our model differs from Miwa et al.’s in three ways: (1) we made stricter assumptions about the effects of age, period, and cohort; (2) we did not consider the effect of salt reduction as a separate effect; and (3) we investigated a different disease (e.g., total cardiovascular disease versus cerebrovascular disease only) and age range (e.g., 20–69 years versus 20–79 years). We focused on individuals aged 69 years or younger because of data availability constraints. An age–period–cohort analysis of stroke mortality reported that the age-adjusted stroke mortality attributable to high salt intake declined by 5% per year from 1990 to 2016 in Japan [31]. The study showed the actual decline in the trend of stroke mortality attributable to high salt intake, while our study showed the discrepancy between the actual cardiovascular mortality rate and that assuming less or no salt-intake reduction.

Relative to the dramatic decline in total deaths and cardiovascular deaths in the late 20th century, the estimated impact of cardiovascular mortality reduction does not appear considerable. However, the effect of salt reduction—which prevented up to 420,000 estimated premature deaths—is comparable to the overall deaths by traffic accidents (approximately 600,000 deaths) during the same period [32]. We assert that consistent efforts to encourage salt-intake reduction as a national nutrition policy, in parallel with the shift from salted to refrigerated storage due to the increasingly widespread use of refrigerators, contributed to salt-intake reduction. Although separating the effect of nutrition policy from that of other environmental factors (e.g., the widespread use of refrigerators) is challenging, examining regional variations in salt reduction campaigns and salt-intake reduction trends may help to speculate on the effect of nutrition policy on lowering salt consumption. Furthermore, this model may help to quantify the impact of future salt-intake reduction on cardiovascular mortality risk.

This study had several limitations. First, the degree of cardiovascular mortality risk reduction due to salt intake was based on a meta-regression study. Moreover, the ratio of period effect to cohort effect was estimated from an assumption. Although the use of parameters from the study was essential to distinguish the impact of salt-intake reduction from that of other temporal changes affecting mortality, our study results may have been sensitive to the changes in these parameters. Second, we assumed that cardiovascular mortality as a proportion of total mortality is the same for all ages within a period because we could not identify information on age-specific cardiovascular mortality by period. Furthermore, we did not assume that the association between salt intake and cardiovascular mortality could be modified by age or other factors. Third, the effect of salt-intake reduction on non-cardiovascular mortality was not considered. In a Japanese cohort study, salty foods (e.g., pickled vegetables and salted fish roe) were reported as risk factors for cancer—especially gastric cancer [33]. Interestingly, the amount of salt intake was not directly associated with the incidence of gastric cancer. We were not able to model the link between salt intake and mortality from other diseases; therefore, we did not take it into consideration, whereas the decrease in salt intake over time could have led to a reduction in gastric cancer mortality. Fourth, the measurements of salt intake used in the underlying studies might not have been optimal, considering that the gold standard of sodium intake is multiple 24 h urine collections. For example, the dietary intake survey of the National Nutrition Surveys and the National Health and Nutrition Surveys adopted semi-weighted household dietary records [27,28]. Some studies included in the meta-regression analysis [30] relied on a food-frequency questionnaire [34], whereas others used a single 24 h urine collection [35]. The coefficients used in this study might, therefore, have been biased toward the null due to salt-intake measurement errors.

## 5. Conclusions

We established a system dynamics model based on an age–period–cohort analysis to estimate the effect of salt-intake reduction on cardiovascular mortality in Japan from 1950 to 2017. Assuming no change in salt intake over the entire period, we estimated approximately 298,000 excess premature deaths of men and 118,000 excess premature deaths of women throughout the study period. The model may help to estimate the effect of salt-intake reduction, partly driven by nutrition policy, in the postwar period in Japan.

## Figures and Tables

**Table 1:** Sources of published data used in the model.

How We Used the Data	Variable Name in the Model	Explanation of Variable	Data Source
Exogenous actual values	Population initials	Population by sex and year as of 1950 for the cohort	Population Estimates [23]
	Annual birth count	Annual births in 1950–2017	Vital Statistics [24]
	Mortality rate table	Mortality rate for each age group in 1950	Life table in Japanese Mortality Database [25]
	CVD mortality proportion table	Proportion of cardiovascular mortality within the overall mortality in 1950–2017	Vital Statistics [24]
	Salt-as is	Salt intake for each age group in 1950–2017	National Nutrition Surveys in 1973–2002 and National Health and Nutrition Surveys in 2003–2017 [26]
	Salt20yo	Salt intake at the age of 20 years old for each cohort	National Nutrition Surveys in 1973–2002 and National Health and Nutrition Surveys in 2003–2017 [26]
Calibration	Mortality rate	Mortality rate for each age group in 1950–2017	Life table in Japanese Mortality Database [25]

## Data Availability

The data presented in this study are available in the Supplementary Figure S1.

## Supplementary Materials

The following supporting information can be downloaded at: https://www.mdpi.com/article/10.3390/nu14183747/s1, Figure S1: The yearly trend of average salt intake in adults in Japan. From the National Nutrition Surveys in 1973–2002 and the National Health and Nutrition Surveys in 2003–2017.
