# Supplementary material for: Estimation of the Effect of Salt-Intake Reduction on Cardiovascular Mortality Decline between 1950 and 2017 in Japan: A Retrospective Simulation Study
Source: Nutrients. 2022 Sep 10;14(18):3747. doi: 10.3390/nu14183747 (PMC9506165; doi:10.3390/nu14183747)
Supplement: Supplementary file 1 [file nutrients-14-03747-s001.zip › nutrients-1898448-supplementary.pdf]

nutrients MDPI

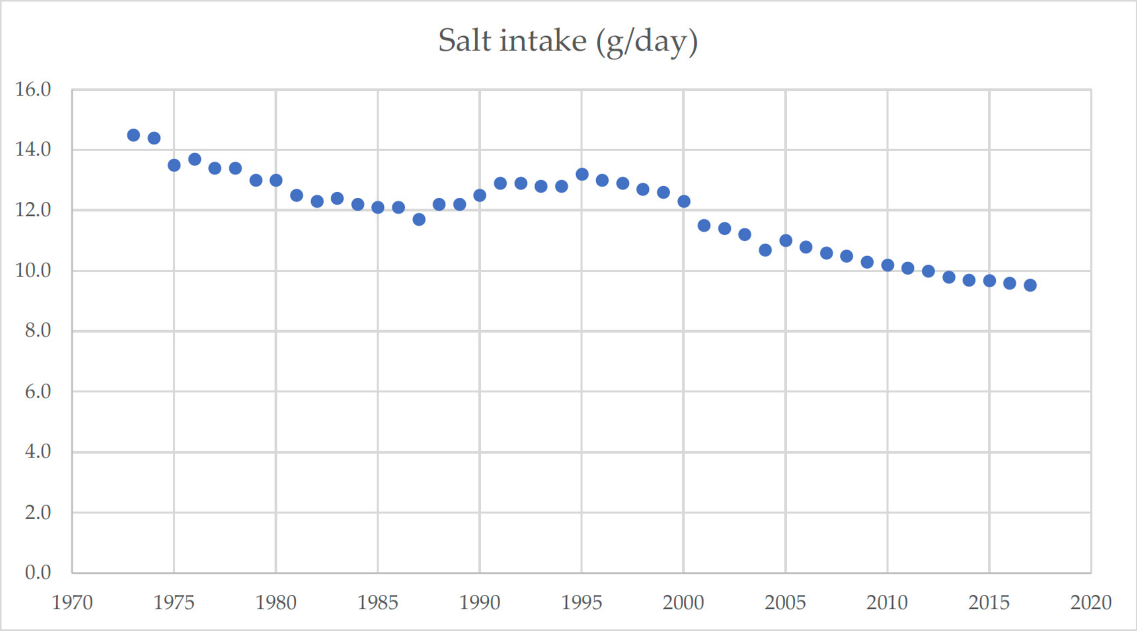

Supplementary Figure S1. The yearly trend of average salt intake in adults in Japan. From the National Nutrition Surveys in 1973–2002 and the National Health and Nutrition Surveys in 2003–2017 [1]. The bullets represent the averages of salt intake by year in the surveys.

1. Ministry of Health, Labour and Welfare. The National Health and Nutrition Survey. Available online: https://www.mhlw.go.jp/bunya/kenkou/kenkou_eiyou_chousa.html (accessed on 5 September 2022).
